# A MUSIC AND MINDFULNESS INTERVENTION FOR PERSONS WITH DEMENTIA AND THEIR CAREGIVERS

**DOI:** 10.1093/geroni/igz038.3436

**Published:** 2019-11-08

**Authors:** Punam G Risal, Debra J Dobbs, Soomi Lee, Jennifer Bugos, Sheikh I Ahamed, William Monaco, William Patterson, Hongdao Meng

**Affiliations:** 1 School of Aging Studies, University of South Florida, Tampa, Florida, United States; 2 University of South Florida, Tampa, Florida, United States; 3 Marquette University, Milwaukee, United States; 4 Digital Media Innovations and eLearning Enterprises, Tampa, Florida, United States

## Abstract

The number of older adults with Alzheimer’s Dementia is projected to increase by 28.6% in Florida by 2025. Cost-effective non-pharmacological interventions targeting both persons with dementia (PWD) and their family caregivers (FCs) are urgently needed. This small pilot study tested the feasibility of a music and mindfulness intervention in an assisted living community (ALC) in Tampa, FL. Methods: We used a mixed-methods single group pre-post study design. To date, a total of 5 PWD and 3 FCs were enrolled, and all completed the 4-week intervention (12 sessions for PWD and 4 sessions for FCs). Outcome measures include behavioral symptoms and sleep quality for PWD, and mindfulness, sleep quality and perceived stress for FCs. Outcome data were collected via survey questionnaires and focus group interviews (FCs and staff). Results: Preliminary analysis showed that the intervention is well-received by PWD, FCs, and ALC staff. PWD participants experienced a reduction in behavioral symptoms post-intervention (Cohen’s d=0.33). FC participants reported increased mindfulness and reduced stress. In focus group interviews, both FCs and staff corroborated the positive findings in residents’ mood and behaviors. Barriers to large scale implementation include intervention time commitment (ALC staff) and scheduling difficulties due to work (FCs). Both FCs and ALC staff recommended the continuation of the intervention and making it available to all memory care residents. Based on the initial results, additional testing of the intervention is currently in progress (10 PWD) and updated results will be reported. Implications for conducting community-based non-pharmacological interventions will be discussed.

